# Cover

**DOI:** 10.4269/ajtmh.1121_scover

**Published:** 2025-01-07

## Abstract

Malaria data review with National Malaria Control staff in a health facility, Rural District, Sierra Leone. *Photo Credit: Stanley Ifeany*

Supervision of field team during digital data collection of seasonal malaria chemoprevention data, Niger. *Photo Credit: Celestin Kwabeng*

Field worker collecting SMC data in household using digital tool, Niger. *Photo Credit: Celestin Kwabeng*

